# Response of a chemo-resistant triple-negative breast cancer patient to a combination of p62-encoding plasmid, Elenagen, and CMF chemotherapy

**DOI:** 10.18632/oncotarget.27323

**Published:** 2020-01-21

**Authors:** Dmitry M. Ponomarenko, Vladimir L. Gabai, Albert A. Sufianov, Sergey I. Kolesnikov, Alexander M. Shneider

**Affiliations:** ^1^Irkutsk State Medical Academy of Postgraduate Education, Irkutsk Regional Cancer Dispensary, Irkutsk, Russian Federation; ^2^CureLab Oncology, Inc, Dedham, MA, USA; ^3^Department of Biochemistry, Boston University School of Medicine, Boston, MA, USA; ^4^Federal Center of Neurosurgery, Tyumen, Russian Federation; ^5^Sechenov First Moscow State Medical University, Moscow, Russian Federation; ^6^Russian Academy of Sciences, Moscow, Russian Federation; ^7^Lomonosov Moscow State University, Moscow, Russian Federation; ^8^Research Center of Family Health and Reproduct ion Problems, Irkutsk, Russian Federation; ^9^Department of Molecular Biology, Ariel University, Ariel, Israel

**Keywords:** cancer immunotherapy, cancer chemotherapy, p62 plasmid, cancer vaccine

## Abstract

Triple-negative breast cancers are often characterized by aggressive behavior and short clinical course once they become chemotherapy-resistant. We describe below a patient who has shown a response to combination of chemotherapy with Elenagen, a plasmid encoding p62. Elenagen was tested in a previous phase I/II study in patients with refractory solid tumors and shown to be safe. Also, plasmid ability to halt tumor progression and restore sensitivity to chemotherapy was found. Preclinical data supports effects on tumor grade and change the tumor’s microenvironment in spontaneous canine breast cancers. We describe here a 48-year old female with triple-negative and BRCA1/2-negative breast cancer who had a primary resistance to chemotherapy and negative dynamics despite the use of multiple lines of treatments. Elenagen was applied intramuscularly at a dose of 1 mg weekly in combination with standard chemotherapy scheme CMF (cyclophosphamide, methotrexate, fluorouracil). In this patient we observed partial tumor regression (by 33%) and 19 weeks of progression-free survival. This first observed objective response to a combination of Elenagen with chemotherapy demonstrates that even in heavily pretreated chemo-resistant triple-negative tumor, the addition of Elenagen to a chemotherapy regimen can cause an objective response and increase in progression-free survival. Such a regimen is worthy of further study in a larger number of patients.

## INTRODUCTION

Breast cancer is one of the most common tumors, and every year more than two million women in the world are diagnosed with it (https://www.wcrf.org/dietandcancer/cancer-trends/breast-cancer-statistics). Most breast cancers are not hereditary: frequency of hereditary predisposition of breast cancer is about 25% of all cases. Among the subtypes of breast cancer, triple-negative breast cancers (TNBC) which does not express estrogen, progesterone and HER2 receptors account for 15% of cases and are characterized by low levels of differentiation of tumor cells and rapid aggressive growth [1, 2].

For such receptor-negative cancers, chemotherapy is the method of choice, but there is no single standard approach [3]. Poly-chemotherapy is characterized by an increase in the number of objective responses and time to progression, while mono-chemotherapy is associated with lower toxicity [4, 5]. Given that the results of overall survival are comparable when using mono - or poly-chemotherapy, in patients without rapid progression, preference should be given to mono-chemotherapy. With TNBC, the greatest efficiency is demonstrated by taxanes and anthracyclines [6, 7].

Immunotherapy of cancer has become one of the leading new methods of treatment. In anti-tumor immunotherapy there are two main approaches. The first is the creation of adaptive antitumor immunity against tumor-specific antigens, i.e. stimulating in patient's body more T-and B-lymphocytes against targets mainly represented in cancer cells, but not in other parts of the body. This direction, in particular, includes DNA vaccines [8, 9]. Another approach is to change the intra-tumor environment and reduce its immunosuppressive properties, and for this the Nobel Prize was awarded in 2018 [10, 11]. In addition, it became clear that the classical chemotherapy regimens, originally created in the paradigm of selectively killing of rapidly dividing cells, also significantly act through stimulation and/or modulation of the immune response [12, 13] and therefore can be considered as some sort of immunotherapy as well. It can be expected that the increasing role of the immune mechanism in the action of chemotherapy regimens will be revealed in the coming years.

Elenagen is a plasmid DNA encoding protein p62/SQSTM. p62 is directly involved in tumor transformation as a regulator of autophagy, an inducer of anti-oxidant proteins, and a modulator of mitotic transit and genomic stability [14, 15, 16]. Initially, the use of the p62 encoding vector was proposed as a classical DNA vaccine, based on the fact that p62 is overexpressed in a wide range of cancers in humans and mouse models [17, 18]. However, it soon became clear that the effect of Elenagen may also be based on its ability to reduce chronic inflammation, manifested in animal models of osteoporosis, metabolic syndrome, and age-related macular degeneration [19–21].

Recent data obtained on spontaneous breast cancer in dogs demonstrates that the use of Elenagen can drastically change the internal structure of the tumor, which can make the tumor more susceptible to therapeutic effects of anticancer treatment (Venanzi, submitted).

Elenagen shows antitumor activity in rodent cancer models and spontaneous tumors in dogs [17, 22], as well as in the phase I/IIa study in patients with disseminated solid tumors that have exhausted standard therapies. Elenagen demonstrated a good safety profile and the ability to stop tumor growth [23].

We present here a case of a robust response to Elenagen in a patient with metastatic TNBC who has been steadily progressing after multiple lines of chemotherapy.

## CLINICAL CASE

A 46-year old female was diagnosed with breast cancer in September 2016. The same month, a radical resection of the right breast with lymphadenectomy was performed. The immuno-morphological study identified invasive unspecified breast cancer G3, with negative status of ER, PR and HER2. Ki67 proliferation index was very high, about 80%. The tumor was staged as pT1pN1M0. Molecular genetic testing of BRCA1 and BRCA2 genes revealed no mutations.

The first course of adjuvant chemotherapy was performed from Nov 2016 to Apr 2017 (Table 1), however, immediately after its completion, a relapse in the remaining part of the breast was diagnosed. A right-sided mastectomy was performed in May 2017 followed by a biopsy of supraclavicular lymph nodes in June 2017; the progression of triple-negative breast cancer was established according to the results of morphological studies. Afterwards, 5 additional rounds of chemotherapy using different drugs and their combinations were performed, but the cancer was unresponsive and progressing (Table 1).

**Table 1:** Lines of chemotherapy applied for treatment of the patient

	Dates	Drug scheme	Result
1	Nov 2016- Apr 2017	Adryamicin+cyclophosphamide (x4), paclitaxel+carbloplatin (x4)	Local recurrence
2	June-Aug 2017	Bevacizumab+capetacibine	Progression
3	Sept-Oct 2017	Eribulin (x2)	Progression
4	Nov-Dec 2017	Gemcitabine+cisplatinum	Progression
5	Jan-Feb 2018	Docetaxel (x2)	Progression
6	Feb-Apr 2018	Vinorelbin+capetacebin	Progression
7	Apr-Aug 2018	Elenagen+cyclophosphamide+methotrexate+fluorouracil	Partial response

**Figure 1:**
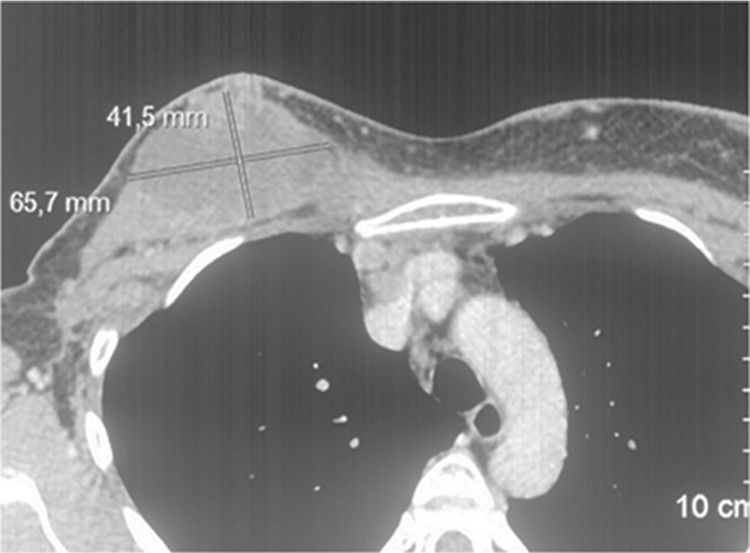
MSCT before the treatment with Elenagen and CMF chemotherapy (the lesion in chest soft tissue in upper left end is marked)

The last chemotherapy regimen before Elenagen treatment, the combination of capecitabine and vinorelbine, was started on Feb 2018 (Table 1). According to the results of the clinical evaluation and MSCT (multi-slice computer tomography) in April 2018, there was a progression of the tumor with multiple lesions of the lymph nodes of the upper mediastinum, right and left axillary, parasternal, supra - and subclavian. Also, there was a soft tissue formation of the chest wall with dimensions of 65,7x42,5 mm and multiple skin metastases and infiltrative lesions of the skin with tumor lymphangitis (Figure 1, Supplementary Figure 1). Due to its inefficiency, this chemotherapy with capecitabine and vinorelbine was cancelled.

Elenagen was started on Apr 2018 along with a standard CMF chemotherapy regimen: cyclophosphamide 600 mg/m^2^ i.v., methotrexate 40 mg/m^2^ i.v., fluorouracil 600 mg/m^2 ^i.v., days 1st and 8^th^, 2-wk interval between courses. Elenagen was injected intramuscularly at a dose of 1 mg weekly, regardless of the timing of the chemotherapy administered. In general, there was a satisfactory tolerability of therapy. Reported adverse effects were: nausea grade 1-2 CTCE, which is usual for courses of the chemotherapy; leucopenia and neutropenia up to grades 2-3, occasionally requiring a postponement of the administration of the chemotherapy; some short-term pain in the projection of the affected lymph nodes (grade 2) 7 weeks from the start of therapy.

**Figure 2:**
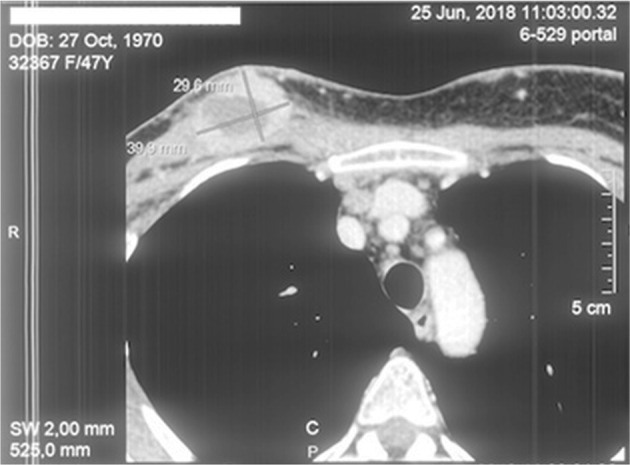
MSCT two months after Elenagen and CMF chemotherapy treatment The lesion in chest soft tissue in upper left end is decreased in size.

A positive dynamic was recorded in June 2018 after two months of Elenagen-CMF treatment. It included improving clinical symptoms, reducing skin itching in places of skin lesions, reducing the area of the wound surface and the number of wound discharge, and reducing the size of visual formations and hyperemia of the affected skin (Supplementary Figure 1). According to MSCT data, there was a decrease in the size of the sum of the diameters of targeted foci by 33%, and there were no new manifestations of the tumor, thus the tumor response is regarded as a partial regression (Figure 2, Supplementary Figure 1). Therefore, it was decided to continue CMF in combination with Elenagen immunotherapy.

After two more months (Aug, 2019), control examination revealed tumor progression due to the resumption of growth of previously existing tumor foci (not shown). Based on this, it was decided to change the chemotherapy regimen to palitaxel/carboplatin with weekly administration of Elenagen, however, with examination in October 2018, the progression of the tumor, the growth of existing foci, and the emergence of a new liver metastasis were revealed. The patient was transferred to symptomatic palliative care and died 3 months later.

## DISCUSSION

We describe a clinical case of using plasmid DNA, encoding р62/SQSTM1 protein, Elenagen, in a patient with metastatic TNBC, whose tumor was progressing after treatment with multiple lines of chemotherapy. The patient didn’t react to any “classicalˮ cytotoxic therapy, but when Elenagen was added she achieved a partial regression of the tumor and progression-free survival for 19 weeks.

For triple-negative cancers, chemotherapy is the method of choice, but there is no single standard approach [3]. During chemotherapy, resistance inevitably develops over time. Feasibility of further use of cytostatics is questionable if a disease is progressing after 2-3 consecutive lines of chemotherapy including taxanes and anthracyclines. In the absence of a standard, the optimal tactics for further management of the patient is selected individually, taking into account the general conditions, manifestations of toxicity and preferences of the patient. Thus, new approaches, drugs, and therapeutic regimens are urgently needed.

Germline mutations in *BRCA1* or *BRCA2 (BRCA1/2)* are present in approximately 10% of patients with TNBC which make them sensitive to alkylating agents (e.g. platinum) or PARP inhibitors [1]. In our case, the TNBC patient had no BRCA1/2 mutations, and there was primary resistance to all types of chemotherapy tested. The effect of Elenagen on tumor regression was observed when combined with CMF chemotherapy although CMF by itself cannot be considered today as an option for a highly effective treatment. However, we observed an objective response (partial tumor regression), and the progression-free survival of 19 weeks. This result was achieved after the patient’s history of different treatment options using monotherapy and combinations of anthracyclines, cyclophosphamide, taxanes, platinum, bevacizumab, eribulin, vinorelbin and capecitabine (Table 1). At the same time, with all the previous therapies, there was no objective response and there was a continuous progression of the tumor. Thus, apparently Elenagen may restore/increase tumor sensitivity to chemotherapy.

There may be at least two mechanisms to explain the observed effect of Elenagen with CMF. The first is that chemotherapy, killing immune-suppressor cells, increases the immune response to Elenagen as a cancer vaccine encoding p62 as a tumor-specific antigen. Because of this, it seems promising to combine the known chemotherapy with drugs that reduce intra-tumor immunosuppression [24]. The second is that Elenagen treatment, via changing the microenviroment (e.g. decreasing inflammation), increases the cytotoxic response to anticancer drugs (e.g., by increasing their delivery [25]. The degree of effectiveness of such combinatory schemes can be quite synergistic.

This case demonstrates the feasibility of further studying, in a larger number of patients, the application of Elenagen concomitantly with chemotherapy to treat heavily pretreated chemo-resistant triple-negative breast cancer patients.

## SUPPLEMENTARY MATERIALS AND FIGURES



## Abbreviations

MSCT: multispiral computed tomography
TNBC: triple-negative breast cancer
