# Supplementary material for: Response of a chemo-resistant triple-negative breast cancer patient to a combination of p62-encoding plasmid, Elenagen, and CMF chemotherapy
Source: Oncotarget. 2020 Jan 21;11(3):294–9. doi: 10.18632/oncotarget.27323 (PMC6980632; doi:10.18632/oncotarget.27323)
Supplement: Supplementary file 1 [file oncotarget-11-294-s001.pdf]

Response of a chemo-resistant triple-negative breast cancer patient to a Combination of p62-encoding plasmid, Elenagen, and CMF chemotherapy

SUPPLEMENTARY MATERIALS

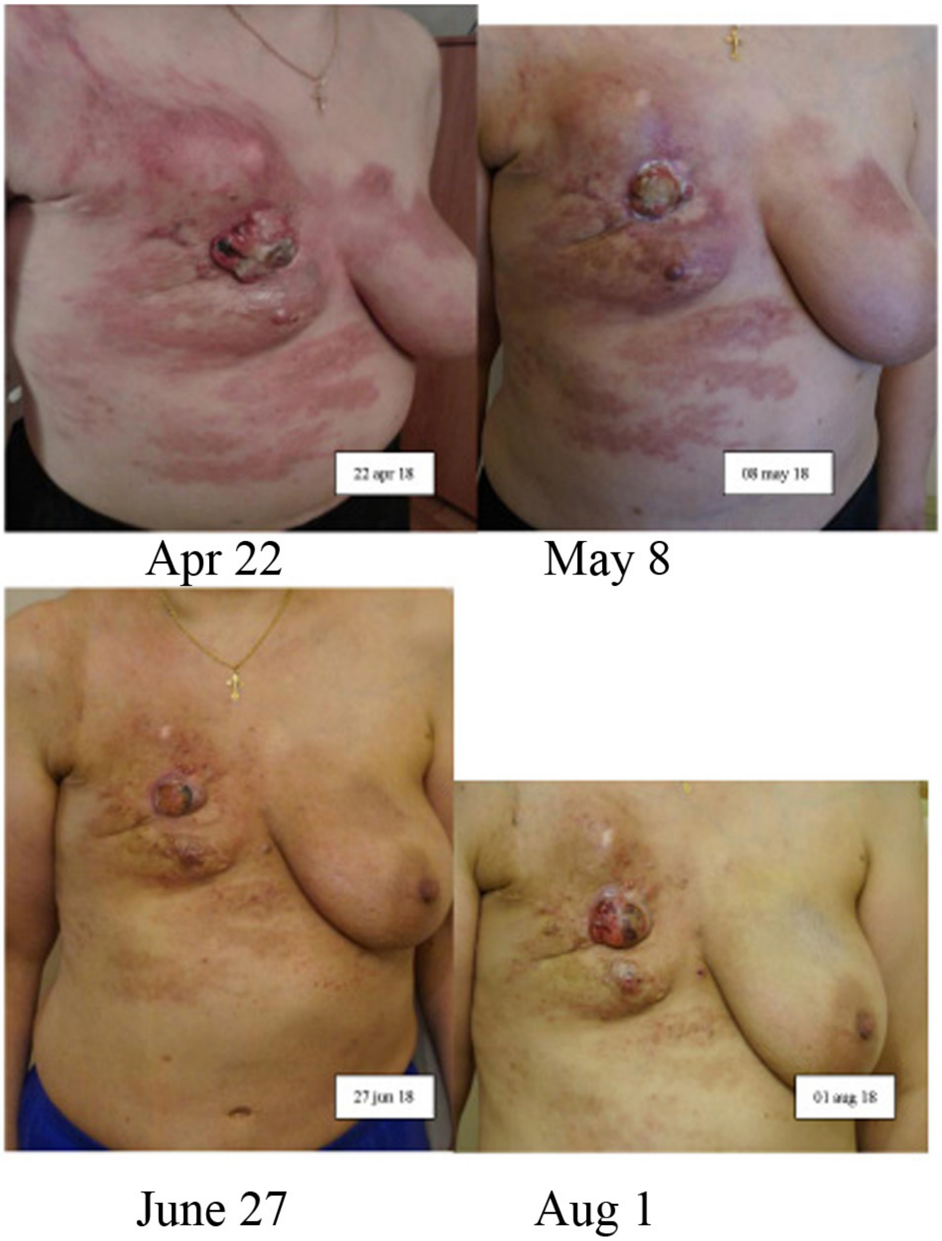

Supplementary Figure 1: Dynamics of tumor growth and skin lesions before and during treatment with Elenagen+ CMF chemotherapy. Apr 22, 2018 – before treatment, May 2- Aug 1 – during treatment.
